# DNA barcoding, identification, and validation of the pufferfish (Order: Tetraodontiformes) in China coastal waters

**DOI:** 10.1002/ece3.10944

**Published:** 2024-02-08

**Authors:** Kaiying Liu, Hongyue Sun, Xin Zhao, Chaoqun Wang, Changting An, Ang Li, Shufang Liu, Zhimeng Zhuang

**Affiliations:** ^1^ National Key Laboratory of Mariculture Biobreeding and Sustainable Goods, Yellow Sea Fisheries Research Institute Chinese Academy of Fishery Sciences Qingdao Shandong China; ^2^ College of Fisheries and Life Science Shanghai Ocean University Shanghai China; ^3^ College of Fisheries Zhejiang Ocean University Zhoushan Zhejiang China; ^4^ Function Laboratory for Marine Fisheries Science and Food Production Processes Laoshan Laboratory Qingdao Shandong China

**Keywords:** DNA barcoding, morphological characteristics, phylogeny, species identification, synonym, Tetraodontiformes

## Abstract

The order Tetraodontiformes are one of the most unique groups of teleostean fish, exhibiting highly derived and greatly diversified phenotypes. It is a difficult task for both professionals and nonprofessionals to accurately identify these species only according to morphological characteristics. DNA barcoding can identify species at the molecular level to overcome the limitations of morphological classification. In this study, we collected 616 specimens of pufferfish from the coastal waters of China. According to the morphological characteristics, they were preliminarily identified as 50 species. Further analysis using DNA barcodes identified these specimens as 46 species, belonging to 23 genera, 6 families. According to the species classification results of DNA barcoding, the three species identified by morphology as *Takifugu pseudommus*, *Takifugu chinensis*, and *Takifugu rubripes* should be the same species. Similarly, *Lagocephalus wheeleri* is the synonym of *Lagocephalus spadiceus*. Another important discovery of DNA barcoding analysis is that there are closer interspecific genetic distances within the genus *Takifugu*. If *T. rubripes*, *T. pseudommus*, and *T. chinensis* are taken as one species, the average interspecific to intraspecific genetic distance ratio of *Takifugu* is only 6.21 times, which does not reach the DNA barcoding threshold of more than 10 times proposed previously. Although the interspecific genetic distance in the genus *Takifugu* is relatively small, each species can be clustered into independent clades in the NJ tree. In conclusion, this study not only found that there are synonymous phenomena in the order Tetraodontiformes but also provided molecular evidence for the valid species names of *Takifugu rubripes* and *Lagocephalus Spadiceus*. The results can provide reliable DNA barcoding information for the identification of pufferfish species, help solve the problem of classification confusion in this order, and provide technical support for the identification of the original components of related commodities on the aquatic product market.

## Graphical abstract

In this study, 616 samples of Tetraodontiformes were identified by using morphological characteristics and DNA barcoding technology. It provided molecular evidence for clarifying the valid species names of *L. spadiceus* and *T. rubripes*. The results can provide reliable DNA barcoding information for the identification and classification of Tetraodontiformes and also provide technical support for the development and utilization of puffer fish resources and the identification of the original components of related commodities on the aquatic product market.

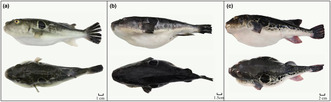

## 1 INTRODUCTION

The latest record from Fishbase (https://www.fishbase.de/) shows that the order Tetraodontiformes comprises approximately 446 species overall, belonging to 106 genera and 10 families. According to the records of “Fauna Sinica,” there are 131 species in 61 genera of 10 families in China (Su & Li, 2002), of which only one species is freshwater pufferfish. Tetraodontiformes are widely distributed along the coast of China and occupy an important economic position in Chinese fishery (Su & Li, 2002). Among them, the pufferfish of three genera *Thamnaconus*, *Takifugu*, and *Lagocephalus* are with high economic value.

The pufferfish have a long history of consumption in China and Japan, and it is regarded as a precious aquatic product for its high protein content and delicious taste. Although pufferfish have a delicious taste and high economic value, many of them contain toxic tetrodotoxin (TTX) in the ovary, liver, kidney, eyes, and blood (Wu & Chen, 1981). If mishandled or ingested, people can become poisoned or even die. Therefore, it is urgent to screen the species of edible pufferfish to avoid poisoning caused by ingestion of toxic pufferfish. The taxonomy and identification of pufferfish is the key to solving the above problems.

In the traditional taxonomy, the classification and identification of Tetraodontiformes are mainly based on the morphological characteristics of stripes, spots, body color, mouth shape, etc. However, the morphology of closely related species of this order is very similar and difficult to distinguish (Chen & Zhang, 2015). Whether professionals or non‐professionals, it is an extremely difficult task to identify pufferfish only according to their morphological characteristics. So far, the phenomenon of synonymous species in the order Tetraodontiformes is not uncommon (Cui et al., 2005; Liu et al., 1999; Matsuura, 2010; Reza et al., 2008; Sakai et al., 2021; Song et al., 2001), and the classification and identification of some species are relatively confused, which makes it difficult to accurately count the number of species in this order (Chen & Zhang, 2015; Su & Li, 2002). Moreover, most of the commodities of the pufferfish in the markets have been processed and lost their original morphological characteristics, which makes it impossible to identify species. It is evident that there is an urgent need for molecular identification of pufferfish species. Due to the ability of DNA barcoding, we can identify species at a molecular level, and the classification results are not affected by the developmental stage and individual morphological integrity of fish, as well as the experience of taxonomists (Hebert et al., 2003). If the morphological classification of species is combined with DNA barcoding technology for species identification, the results of species classification identification will be more objective and reliable.

At present, there have been many reports on the molecular taxonomy study of pufferfish. However, these studies only focused on a few genera or several species of the order Tetraodontiformes (Chen et al., 2019; Elmerot et al., 2002; Ishizaki et al., 2006; Reza et al., 2008; Santini et al., 2013; Song et al., 2001; Zhang & He, 2008), lacking systematic taxonomic study on the species of the entire order. Therefore, it is urgent to carry out research on DNA barcoding classification and identification of the pufferfish, to clarify the synonyms and cryptic species of this order.

In order to solve the difficulty of morphological classification of Tetraodontiformes, this study used DNA bar code technology and morphological methods to identify 616 samples of Tetraodontiformes collected from the coast of China, clarified the synonyms of some species of this order, and confirmed the effective species names of these species Building a perfect classification system of DNA barcode for the order Tetraodontiformes, will not only enrich the database of fish DNA barcode but also promote the rapid development of pufferfish taxonomy and systematics and provide technical support for aquatic product quality safety and trade supervision. The results of this study not only provide a basis for the taxonomic and phylogenetic research of Tetraodontiformes but also provide technical support for market supervision of pufferfish and their processed products.

## 2 MATERIALS AND METHODS

### 2.1 Sample collection

Since 2011, we have collected a total of 616 specimens of pufferfish from the coastal waters of China (Table 1). Samples collection and use comply with the relevant regulations of the Institutional Animal Care and Use Committee.

**TABLE 1:** Sample collection information and identification results.

Voucher no.	GenBank accession no.	Haplotype	Number of samples	Morphological identification	Molecular identification	BLAST result in NCBI	BLAST in BOLD	Family
YSFRI023–YSFRI026	OQ700252–OQ700255	Hap (13–15)	4	*Balistoides conspicillum*	*B. conspicillum*	*B. conspicillum* (99.23%–100%)	*B. conspicillum* (99.38%–100%)	Balistidae
YSFRI028	OQ700257	Hap17	1	*Canthidermis maculata*	*C. maculata*	*C. maculata* (99.19%–100%)	*C. maculata* (98.95%–100%)	Balistidae
YSFRI236–YSFRI241	OQ700465–OQ700470	Hap (69–71)	6	*Melichthys vidua*	*M. vidua*	*M. vidua* (99.39%–99.85%), *M. indicus* (99.07%–99.69%)	*M. vidua* (99.39%–99.85%), *M. indicus* (99.08%–99.70%)	Balistidae
YSFRI279–YSFRI283	OQ700508–OQ700511	Hap79	4	*Rhinecanthus aculeatus*	*R. aculeatus*	*R. aculeatus* (99.42%–100%)	*R. aculeatus* (99.38%–100%)	Balistidae
YSFRI308–YSFRI310	OQ700537–OQ700539	Hap (84–85)	3	*Sufflamen chrysopterum*	*S. chrysopterum*	*S. chrysopterum* (97.46%–99.85%)	*S. chrysopterum* (97.45%–99.85%)	Balistidae
YSFRI311–YSFRI313	OQ700540–OQ700542	Hap (86–87)	3	*Sufflamen fraenatum*	*S. fraenatum*	*S. fraenatum* (98.98%–99.82%)	*S. fraenatum* (98.98%–99.83%)	Balistidae
YSFRI616	OQ700845	Hap171	1	*Xanthichthys auromarginatus*	*X. auromarginatus*	*X. auromarginatus* (98.61%–99.39%)	*X. auromarginatus* (98.6%–99.38%)	Balistidae
YSFRI075–YSFRI093	OQ700304–OQ700322	Hap (24–27)	19	*Diodon holocanthus*	*D. holocanthus*	*D. holocanthus* (99.05%–99.84%)	*D. holocanthus* (99.53%–99.84%)	Diodontidae
YSFRI094–YSFRI102	OQ700323–OQ700332	Hap (28–29)	9	*Diodon hystrix*	*D. hystrix*	*D. hystrix* (99.24%–99.85%)	*D. hystrix* (99.23%–100%)	Diodontidae
YSFRI103–YSFRI107	OQ700332–OQ700336	Hap (30–31)	5	*Diodon liturosus*	*D. liturosus*	*D. liturosus* (99.51%–100%)	*D. liturosus* (99.85%–100%)	Diodontidae
YSFRI001	OQ700230	Hap1	1	*Aluterus monoceros*	*A. monoceros*	*A. monoceros* (98.93%–100%)	*A. monoceros* (97.41%–100%)	Monacanthidae
YSFRI002–YSFRI014	OQ700231–OQ700243	Hap (2–5)	13	*Aluterus scriptus*	*A. scriptus*	*A. scriptus* (99.24%–100%)	*A. scriptus* (99.08%–100%)	Monacanthidae
YSFRI027	OQ700256	Hap16	1	*Cantherhines pardalis*	*C. pardalis*	*C. pardalis* (98.93%–100%), *C. pullus* (99.47%–100%), 2 *C. sandwichiensis* (99.69%–99.70%)	*C. pardalis* (98.8%–100%), *C. pullus* (99.07%–100%), 1 *Stephanolepis setifer* (99.85%)	Monacanthidae
YSFRI029–YSFRI063	OQ700258–OQ700292	Hap (18–19)	35	*Chaetodermis penicilligerus*	*C. penicilligerus*	*C. penicilligerus* (99.54%–100%)	*C. penicilligerus* (99.53%–100%)	Monacanthidae
YSFRI242–YSFRI248	OQ700471–OQ700477	Hap72	7	*Monacanthus chinensis*	*M. chinensis*	*M. chinensis* (97.84%–100%)	*M. chinensis* (97.85%–100%)	Monacanthidae
YSFRI284–YSFRI307	OQ700513–OQ700536	Hap (81–83)	24	*Stephanolepis cirrhifer*	*S. cirrhifer*	*S. cirrhifer* (99.08%–100%), 1 *S. diaspros* (99.69%–100%)	*S. cirrhifer* (99.12%–100%), 1 *S. diaspros* (99.39%–99.69%)	Monacanthidae
YSFRI567–YSFRI582	OQ700796–OQ700811	Hap (153–158)	16	*Thamnaconus hypargyreus*	*T. hypargyreus*	*T. hypargyreus* (99.43%–100%), 2 *T. septentrionalis* (99.85%–100%)	*T. hypargyreus* (99.4%–100%), 4 *T. septentrionalis* (99.85%–100%)	Monacanthidae
YSFRI583–YSFRI605	OQ700812–OQ700834	Hap (159–167)	23	*Thamnaconus modestus*	*T. modestus*	*T. modestus* (99.36%–100%), 3 *T. modestoides* (99.1%–99.85%); 2 *T. septentrionalis* (99.84%–99.85%)	*T. modestus* (99.42%–100%), *T. modestoides* (99.69%–99.85%), 1 *T. septentrionalis* (99.19%–99.84%)	Monacanthidae
YSFRI108–YSFRI114	OQ700337–OQ700344	Hap (32–34)	7	*Lactoria cornuta*	*L. cornuta*	*L. cornuta* (99.81%–100%)	*L. cornuta* (99.35%–100%)	Ostraciidae
YSFRI249	OQ700478	Hap73	1	*Ostracion cubicus*	*O. cubicus*	*O. cubicus* (98.01%–100%), 4 *O. immaculatus* (99.56%–100%)	*O. cubicus* (97.99%–100%), 5 *O. immaculatus* (99.42%–100%)	Ostraciidae
YSFRI250–YSFRI257	OQ700479–OQ700486	Hap (74–77)	8	*Ostracion rhinorhynchos*	*O. rhinorhynchos*	*O. rhinorhynchos* (99.39%–99.42%)	*O. rhinorhynchos* (98.76%–99.54%)	Ostraciidae
YSFRI015–YSFRI017	OQ700244–OQ700246	Hap (6–8)	3	*Arothron hispidus*	*A. hispidus*	*A. hispidus* (96.41%–100%)	*A. hispidus* (97.61%–100%)	Tetraodontidae
YSFRI018	OQ700247	Hap9	1	*Arothron mappa*	*A. mappa*	4 *A. mappa* (99.7%–99.85%), 1 *A. stellatus* (99.56%)	2 *A. mappa* (99.7%–99.85%), 2 *A. stellatus* (99.56%)1 *Tylerius* sp. (100%)	Tetraodontidae
YSFRI019–YSFRI022	OQ700248–OQ700251	Hap (10–12)	4	*Arothron stellatus*	*A. stellatus*	*A. stellatus* (98.34%–99.67%), 1 *A. multilineatus* (99.56%–100%)	*A. stellatus* (98.23%–99.85%)	Tetraodontidae
YSFRI064–YSFRI074	OQ700293–OQ700303	Hap (20–23)	11	*Dichotomyctere nigroviridis*	*D. nigroviridis*	*Tetraodon nigroviridis* (98.22%–99.56%)	*Tetraodon nigroviridis* (98.16%–99.53%)	Tetraodontidae
YSFRI115–YSFRI131	OQ700344–OQ700360	Hap (35–43)	17	*Lagocephalus gloveri*	*L. gloveri*	*L*. *gloveri* (98.54%–100%); *L. cheesemanii* (99.42%–99.85%), 1 *L. spadiceus* (99.69%–99.85%)	*L. gloveri* (98.53%–100%); *L. cheesemanii* (99.42%–100%), 2 *L. spadiceus* (99.69%–100%)	Tetraodontidae
YSFRI132–YSFRI139	OQ700361–OQ700368	Hap (44–49)	8	*Lagocephalus inermis*	*L. inermis*	L. *inermis* (98.19%–99.82%)	*L. inermis* (98.01%–99.82%)	Tetraodontidae
YSFRI140–YSFRI153	OQ700369–OQ700382	Hap (50–53)	14	*Lagocephalus spadiceus*	*Lagocephalus spadiceus*	*L. wheeleri* (99.39%–100%), *L. spadiceus* (97.38%–100%); 2 *L. gloveri* (99.85%–100%)	*L. spadiceus* (97.17%–100%); 2 *L. gloveri* (100%), 1 *L. inermis* (99.81%–100%)	Tetraodontidae
**YSFRI154–YSFRI235**	**OQ700383–OQ700464**	**Hap (50, 53–68)**	**82**	***Lagocephalus wheeleri***	***Lagocephalus spadiceus***	***L. wheeleri* (99.39%–100%), *L. spadiceus* (97.38%–100%); 2 *L. gloveri* (99.85%–100%)**	***L. spadiceus* (97.17%–100%); 2 *L. gloveri* (100%), 1 *L. inermis* (99.81%–100%)**	**Tetraodontidae**
YSFRI258–YSFRI278	OQ700487–OQ700507	Hap78	21	*Pao leiurus*	*P. leiurus*	*P*. sp. (99.84%–100%), 2 *Monotrete leiurus* (98.36%–99.56%), 1 *P. abei* (99.56%)	*P*. sp. (99.84%–100%), *P. cambodgiensis* (98.36%–100%), *P. leiurus* (98.11%–100%)	Tetraodontidae
YSFRI283	OQ700512	Hap80	1	*Sphoeroides pachygaster*	*S. pachygaster*	*S. pachygaster* (97.48%–100%)	*S. pachygaster* (97.46%–100%), 1 *Lagocephalus Lagocephalus*. (99.85%)	Tetraodontidae
YSFRI314–YSFRI323	OQ700543–OQ700552	Hap (88–90, 108)	10	*Takifugu alboplumbeus*	*T. alboplumbeus*	*T. alboplumbeus* (99.13%–100%), *T. niphobles* (98.19%–100%)	*T. alboplumbeus* (98.33%–100%), *T. niphobles* (98.35%–100%), 2 *T. porphyreus* (99.64%–100%)	Tetraodontidae
YSFRI324–YSFRI356; YSFRI358	OQ700553–OQ700585; OQ700587	Hap (91–95,97)	34	*Takifugu bimaculatus*	*T. bimaculatus*	*T. bimaculatus* (99.71%–100%)	*T. bimaculatus* (99.32%–99.66%)	Tetraodontidae
**YSFRI359–YSFRI366**	**OQ700588–OQ700595**	**Hap (98–99)**	**8**	***Takifugu chinensis***	***T. rubripes***	***T. chinensis* (99.55%–100%), 2 *T. pseudommus* (99.56%–99.71%), *T. rubripes* (99.36%–99.84%), 1 *T. flavidus* (99.85%)**	***T. chinensis* (99.51%–100%), 1 *T. pseudommus* (99.51%), *T. rubripes* (99.34%–99.85%), 2 *T. flavidus* (99.85%)**	**Tetraodontidae**
YSFRI357；YSFRI367–YSFRI388	OQ700586；–OQ700596–617	Hap (96, 100–106)	23	*Takifugu flavidus*	*T. flavidus*	*T. flavidus* (99.54%–99.84%), 3 *T. fasciatus* (99.85%–100%)	*T. flavidus* (99.7%–99.84%), 4 *T. fasciatus* (99.85%–100%)	Tetraodontidae
**YSFRI389–YSFRI409**	**OQ700618–OQ700638**	**Hap (88,107)**	**21**	***Takifugu niphobles***	***T. alboplumbeus***	***T. alboplumbeus* (99.13%–100%), *T. niphobles* (98.19%–100%)**	***T. alboplumbeus* (98.33%–100%), *T. niphobles* (98.35%–100%), 2 *T. porphyreus* (99.64%–100%)**	**Tetraodontidae**
YSFRI410–YSFRI435	OQ700639–OQ700664	Hap (109–115)	26	*Takifugu oblongus*	*T. oblongus*	*T. oblongus* (98.45%–100%)	*T. oblongus* (98.26%–100%)	Tetraodontidae
YSFRI436–YSFRI449	OQ700665–OQ700678	Hap (116–119)	14	*Takifugu obscurus*	*T. obscurus*	*T. obscurus* (99.55%–99.85%), *T. fasciatus* (99.56%–99.69%)	*T. obscurus* (99.55%–99.85%), 2 *T. fasciatus* (99.56%)	Tetraodontidae
YSFRI450–YSFRI485	OQ700679–OQ700714	Hap (120–128)	36	*Takifugu ocellatus*	*T. ocellatus*	*T. ocellatus* (99.67%–100%)	*T. ocellatus* (99.55%–100%)	Tetraodontidae
YSFRI486–YSFRI487	OQ700715–OQ700716	Hap129	2	*Takifugu pardalis*	*T. pardalis*	*T. pardalis (*99.42%–100%)	*T. pardalis* (99.7%–100%)	Tetraodontidae
YSFRI488–YSFRI492	OQ700717–OQ700721	Hap (130–132)	5	*Takifugu poecilonotus*	*T. poecilonotus*	*T. poecilonotus* (98.98%–100%), 1 *T. exascurus* (99.56%–99.71%), 1 *T. alboplumbeus* (99.26%–99.71%)	*T. poecilonotus* (99.4%–100%), 2 *T. exascurus* (99.56%–99.71%)	Tetraodontidae
YSFRI493–YSFRI495	OQ700722–OQ700724	Hap (133–135)	3	*Takifugu porphyreus*	*T. porphyreus*	*T. porphyreus* (99.41%–99.71%)	*T. porphyreus* (99.24%–99.71%)	Tetraodontidae
**YSFRI496–YSFRI499**	**OQ700725–OQ700728**	**Hap (99, 136)**	**4**	***Takifugu pseudommus***	***T. rubripes***	***T. rubripes* (99.52%–100%), 2 *T. pseudommus* (99.71%–99.85%), *T. chinensis* (99.54%–100%), 1 *T. flavidus* (99.71%–100%)**	***T. rubripes* (99.5%–100%), 1 *T. pseudommus* (99.67%–99.84%), *T. chinensis* (99.35%–99.67%), 2 *T. flavidus* (99.71%–100%)**	**Tetraodontidae**
YSFRI500–YSFRI526	OQ700729–OQ700755	Hap (99, 136–139)	27	*Takifugu rubripes*	*T. rubripes*	*T. rubripes* (99.52%–100%), 2 *T. pseudommus* (99.56%–99.85%), *T. chinensis* (99.54%–100%), 1 *T. flavidus* (99.71%–100%)	*T. rubripes* (99.5%–100%), 1 *T. pseudommus* (99.35%–99.67%), *T. chinensis* (99.35%–99.67%), 2 *T. flavidus* (99.71%–100%)	Tetraodontidae
YSFRI527–YSFRI528	OQ700756–OQ700757	Hap140	2	*Takifugu snyderi*	*T. snyderi*	*T. snyderi* (100%), *T. stictonotus* (98.5%–100%)	*T. snyderi* (100%), *T. stictonotus* (98.5%–100%)	Tetraodontidae
YSFRI529	OQ700758	Hap141	1	*Takifugu stictonotus*	*T. stictonotus*	*T. snyderi* (98.69%–99.85%)	*T. snyderi* (98.68%–99.85%)	Tetraodontidae
YSFRI530–YSFRI533	OQ700759–OQ700762	Hap (142–144)	4	*Takifugu vermicularis*	*T. vermicularis*	*T. vermicularis* (99.42%–99.71%)	*T. vermicularis* (99.38%–99.71%)	Tetraodontidae
YSFRI534–YSFRI566	OQ700763–OQ700795	Hap (145–152)	33	*Takifugu xanthopterus*	*T. xanthopterus*	*T. xanthopterus* (99.26%–99.85%)	*T. xanthopterus* (99.37%–100%)	Tetraodontidae
YSFRI606–YSFRI614	OQ700835–OQ700843	Hap (168–169)	9	*Torquigener hypselogeneion*	*T. hypselogeneion*	*T. hypselogeneion* (98.27%–100%), *T. flavimaculosus* (97.98%–99.85%), *T. altipinnis* (99.41%–99.55%)	*T. hypselogeneion* (97.77%–100%), *T. flavimaculosus* (96.59%–99.85%), 1 *T. perlevis* (99.7%–99.85%), 1 *T. hicksi* (99.53%99.69%), *T. altipinnis* (99.4%)	Tetraodontidae
YSFRI615	OQ700844	Hap170	1	*Triacanthus biaculeatus*	*T. biaculeatus*	*T. biaculeatus* (99.68%–99.85%)	*T. biaculeatus* (99.69%–99.98%)	Triacanthidae
Total		171	616	50 species	47species			6

*Note*: The bold items in the table refer to samples with differences in morphology and molecular identification.

After preliminary classification according to morphological characteristics, they were preserved in 95% ethanol for subsequent DNA barcoding identification. All voucher specimens are deposited in the National Marine Fishery Biological Germplasm Resource Bank.

### 2.2 Morphological identification

Firstly, the body length, weight, and other measurable traits of the specimen were measured. Further species identification was conducted based on the morphological characteristics of pufferfish described by Chen and Zhang (2015) and Su and Li (2002), including body size, number of fins, stripes, spots, body color, mouth shape, etc.

### 2.3 DNA extraction and quality assessment

DNA was extracted from the muscle of fish by using TIANamp Marine Animals DNA Kit (Tiangen Biotech (Beijing) Co., Ltd.). For detailed steps of DNA extraction, refer to the kit manual. DNA samples were stored at −20°C.

Nano‐300 micro‐spectrophotometer (Allsheng) was used to detect the concentration and purity of DNA samples, and the quality of the DNA samples was detected by 1% agarose gel.

### 2.4 PCR amplification and DNA sequencing

The published universal primers for fish DNA barcodes (Ward et al., 2005) were used for PCR amplification and sequencing, and the length of the amplification product was 707 bp. The primers were as follows:

COIF: 5′‐TCAACCAACCACAAAGACATTGGCAC‐3′; COIR: 5′‐TAGACTTCTGGGTGGCCAAAGAATCA‐3′.

PCR amplification was in total volume of 25 μL, containing 12.5 μL Taq Mixture (2×, vazyme), 2 μL DNA template, 1 μL of each primer (10 μM), and 8.5 μL ddH2O. Thermo cycling was carried out as follows: 94°C for 5 min, 94°C for 30 s, 55°C for 30 s, 72°C for 1 min, cycle 35 times from step 2 to step 4, and finally 72°C for 10 min. PCR products were detected by 1% agarose gel, and the qualified samples were sent to BGI in Qingdao for direct bidirectional sequencing.

### 2.5 Data analysis

The raw sequences were assembled by seqman of Lasergene software package (Burland, 2000; Swindell & Plasterer, 1997) and aligned by Clustal W in BioEdit version 7.0.9 (Hall, 1999). After correction and alignment, the effective sequence length was 687 bp. The haplotypes were analyzed by DNASP version 5.10.01 (Librado & Rozas, 2009). Each haplotype sequence was compared for similarity in NCBI (https://www.ncbi.nlm.nih.gov/) and BOLD (http://www.barcodinglife.org/). The top matches showing ≥98% similarity were used as the preliminary identification results. Then based on Kimura‐2‐parameter (K2P) mode (Kimura, 1980), the genetic distance of different taxonomic levels of Tetraodontiformes was calculated.

*Bothus myriaster* (NC_030365) and *Glyptocephalus stelleri* (NC_060723) were selected as the outgroups, based on the haplotypes of COI gene of Tetraodontiformes, the Neighbor‐joining tree (NJ tree) of Tetraodontiformes was constructed in Mega 7.0 (Kumar et al., 2016).

## 3 RESULTS

### 3.1 Morphological identification

According to the morphological characteristics, 616 samples were identified as 50 species, belonging to 23 genera, 6 families, and 4 Suborder (Table 1). Among them, 612 samples can be identified to species. The remaining four samples are larval and juvenile fish, which can only be identified to genus due to their atypical morphological characteristics.

**FIGURE 1:**
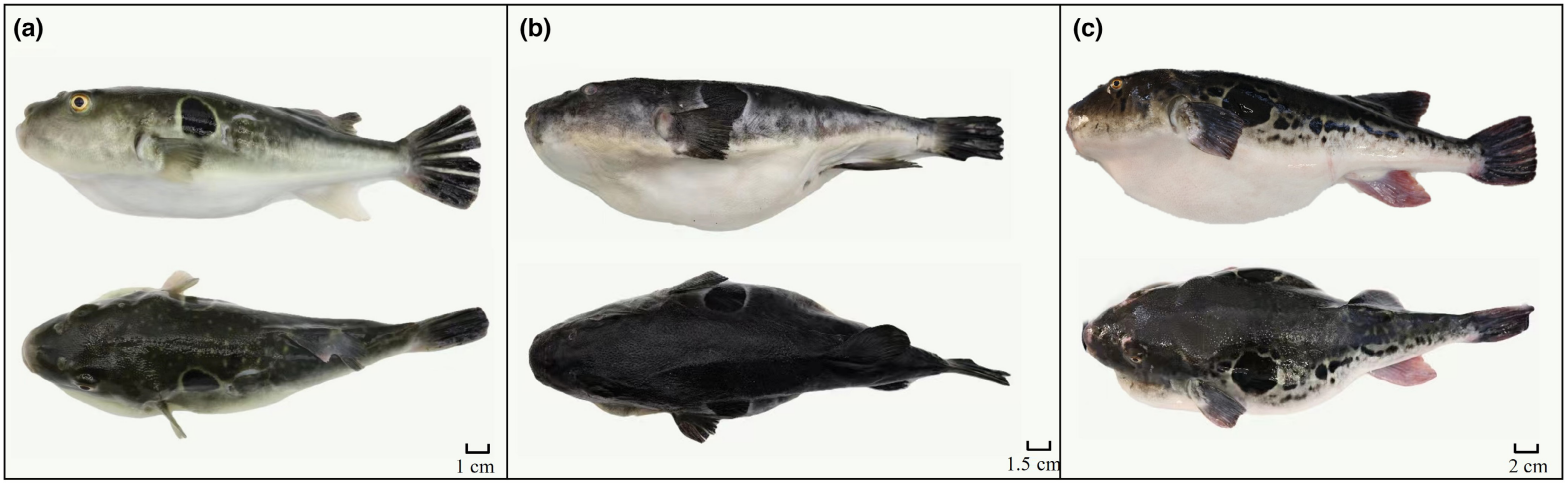
Lateral (left) and dorsal (middle) views of *Takifugu* spp. (a) *T. pseudommus*; (b) *T. chinensis*; (c) *T. rubripes*.

Among the 23 genera of Tetraodontiformes collected in this study, the various species of *Takifugu* genus have very similar morphologies and are difficult to distinguish with the naked eye. For example, the morphologic characteristics of *T. alboplumbeus* and *T. niphobles* are very similar. It is generally believed that *T. niphobles* has obvious chest spots. The chest spots of *T. alboplumbeus* are not obvious, but there are 4–6 dark bands on its back. Out of 616 specimens, 21 were identified as *T. niphobles*. And 10 specimens have the characteristics of *T. alboplumbeus*, but they are not exactly the same as *T. alboplumbeus* because they only present two dark bands. Furthermore, it is also difficult to distinguish *T. rubripes*, *T. pseudommus*, and *T. chinensis*, some studies suggest *T. rubripes* has irregular round black spots and white patterns in front of the caudal fin on the sides of the body, *T. chinensis* has no such black marks while *T. pseudommus* has white spots scattered on a black background on the dorsal and lateral sides of the body (Baek et al., 2018; Reza et al., 2008), as shown in Figure 1. Based on these morphological characteristics, 8 specimens were identified as *T. chinensis*, 4 as *T. pseudommus*, and 27 as *T. rubripes*.

**FIGURE 2:**
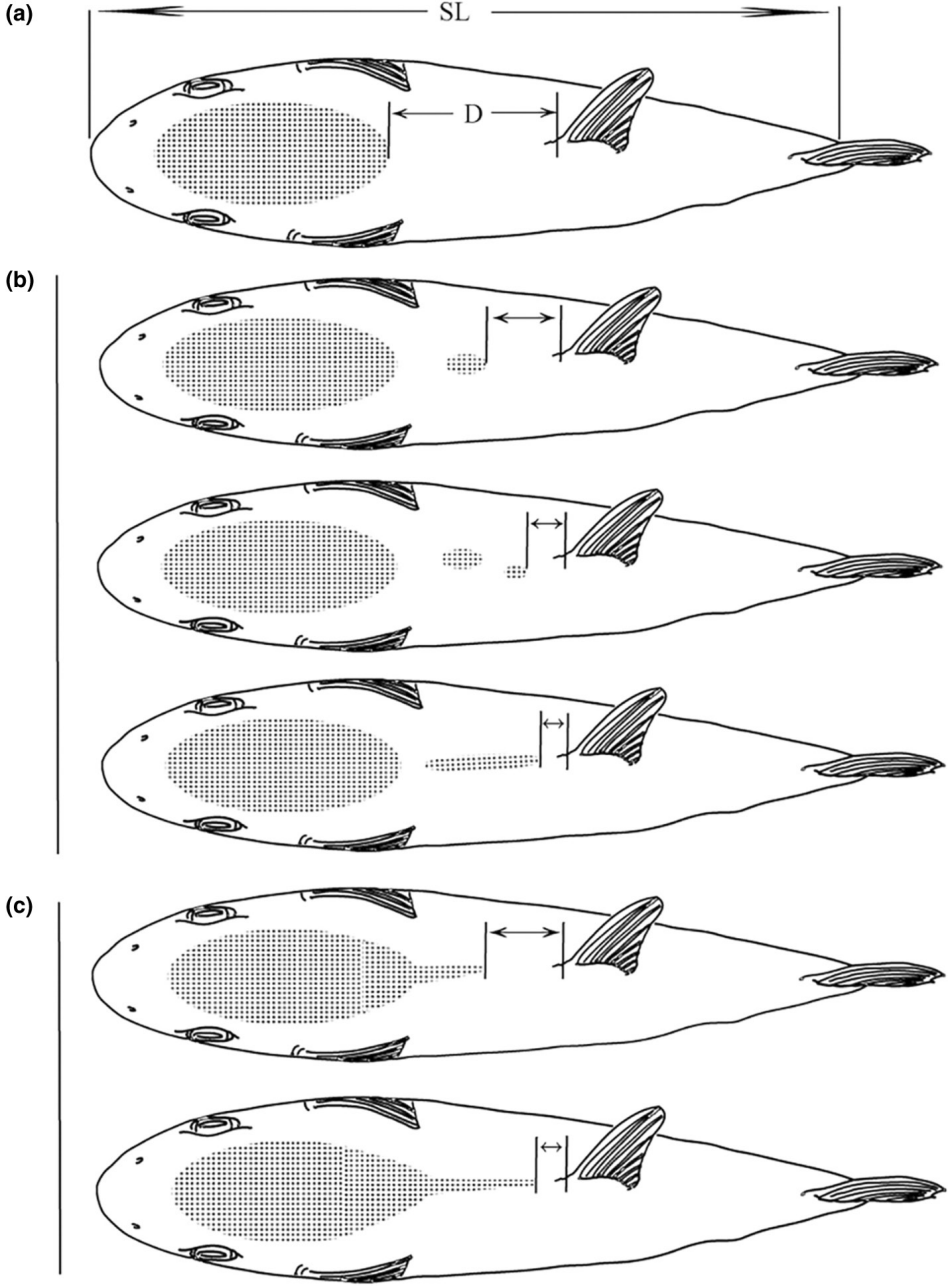
Dorsal spinule patch forms of *Lagocephalus* spp. (Sakai et al., 2021); (a) *Lagocephalus wheeleri*; (b) Intermediate type of *Lagocephalus spadiceus* and *L. wheeleri*; (c) *L. spadiceus*; D, distance from dorsal spinule patch tail to origin of dorsal fin; SL, standard length.

Additionally, *Lagocephalus wheeleri* (Abe, Tabeta & Kitahama, 1984) and *Lagocephalus spadiceus* (Richardson, 1845) can also be distinguished by morphological characteristics. The former has elliptical dorsal spinule patch, while the latter has a rhomboidal patch with a posterior extension. In this study, 82 specimens were identified as *L. wheeleri*, 10 were identified as *L. spadiceus*, and 4 were found to have the intermediate characteristics of these two species, covering discontinuous patch. The above three morphological differences can be seen in Figure 2.

### 3.2 DNA barcoding of the Tetraodontiformes

#### 3.2.1 Characteristics of DNA barcode sequences of Tetraodontiformes

The COI gene sequences of 616 pufferfish were retained with an effective length of 687 bp, consisting of 370 conserved sites, 62 transition sites (si), 43 transversion site (sv), and the si/sv ratio was 1.4. The results of base composition analysis showed: T 27.7%, C 29.9%, A 23.8%, G 18.6%, and the content of A + T was 51.5%, and C + G was 48.5%. Among them, the average G + C content of the first base of the codon was the highest, reaching 56.9%. The sequence analysis results also showed that the COI gene sequences of various species had high similarity with 561 conserved site in 253 sequences in *Takifugu* genus.

#### 3.2.2 Haplotype analysis of DNA barcodes in Tetraodontiformes

Haplotype analysis showed that there were a total of 171 haplotypes in 616 COI gene sequences of Tetraodontiformes. Among them, there were 65 haplotypes in 253 COI gene sequences of genera *Takifugu*. Most noteworthy, the three species of *T. pseudommus*, *T. rubripes*, and *T. chinensis* share the same haplotype Hap 99; Hap 136 is the same haplotype shared by *T.rubripes* and *T. pseudommus*; and Hap 88 is the same haplotype shared by *T. alboplumbeus* and *T. niphobles*. In addition, among the 121 COI gene sequences of genera *Lagocephalus*, there were 36 haplotypes, with the shared haplotype of Hap 50 and Hap 53 for *L. wheeleri* and *L. spadiceus*.

#### 3.2.3 Genetic distance of Tetraodontiformes

Using 50 species identified through morphological identification as taxonomic units, calculate the intra‐ and inter‐species genetic distances of DNA barcodes. The results showed that the intraspecific genetic distance ranged from 0 to 0.009, with an average of 0.003. The interspecific genetic distance ranged from 0.001 to 0.298, with an average of 0.212. The ratio of the average intraspecific to interspecific genetic distance was 70.63. The average genetic distance within the genus was 0.016. The genetic distance between genera ranged from 0.12 to 0.298, with an average of 0.223. The average genetic distance within the family was 0.106. The genetic distance between families ranged from 0.192 to 0.247, with an average of 0.229.

We found that the interspecific genetic distance of several species was very low, far below the threshold of interspecific genetic distance for DNA barcoding. For example, the interspecific genetic distance between *T. alboplumbeus* and *T. niphobles* was only 0.002; between *L. wheeleri* and *L. spadiceus* was only 0.003. Additionally, the interspecific genetic distance of *T. pseudommus*, *T. rubripes*, and *T. chinensis* was 0.001 to 0.002.

Moreover, the results also showed that the species of *Takifugu* have very close genetic relationships. In this genus, the average interspecific genetic distance is only 6.21 times of the average intraspecific genetic distance, if *T. rubripes*, *T. pseudommus*, and *T. chinensis* are taken as one species, and *T. alboplumbeus* and *T. niphobles* are also considered as one species. It does not reach the DNA barcode threshold of more than 10 times proposed by Hebert et al. (2004). Among them, the genetic distance between *T. oblongus* and *T. stictonotus* is the largest, 0.045; and between *T. bimaculatus* and *T. flavidus* is the smallest, only 0.013. Meanwhile, there are several other species in this genus with interspecific genetic distance <0.02 (as shown in Table 2).

**TABLE 2:** The species pairs in *Takifugu* with interspecific genetic distance <0.02.

Species 1	Species 2	Interspecific genetic distance
*T. flavidus*	*T. bimaculatus*	0.0127
*T. flavidus*	*T. obscurus*	0.0196
*T. xanthopterus*	*T. obscurus*	0.0199
*T. xanthopterus*	*T. pardalis*	0.0159
*T. xanthopterus*	*T. poecilonotus*	0.0157
*T. stictonotus*	*T. snyderi*	0.0133

#### 3.2.4 Phylogenetic relationships of Tetraodontiformes

**FIGURE 3:**
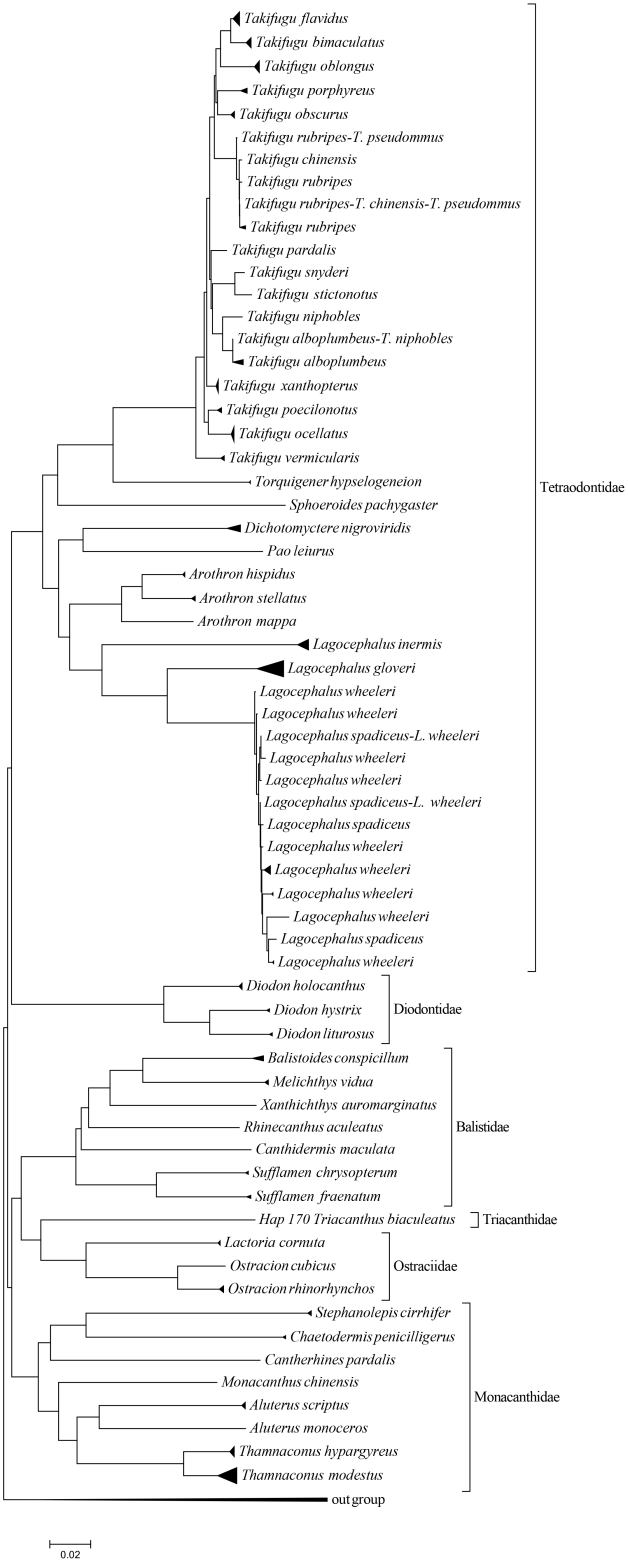
The phylogenetic tree of Tetraodontiformes based on 171 COI gene haplotypes.

The Neighbor‐joining tree was constructed for the order Tetraodontiformes, based on the haplotypes of COI gene. The results indicate that the six families of this order in this study are divided into two branches, namely the Diodontidae and Tetraodontidae families, which converge into one branch, while the Monacanthidae, Balistidae, Ostraciidae, and Triacanthidae families converge into another branch. As expected, in the Neighbor‐joining tree, *T. rubripes*, *T. pseudommus*, and *T. chinensis* clustered into one clade, while *T. alboplumbeus* and *T. niphobles* clustered into one clade, and *L. wheeleri* and *L. spadiceus* also clustered into one clade. This once again confirms the previously proposed hypothesis of synonyms. In addition, we found that although the interspecific genetic distance of the genus *Takifugu* is small, some even <0.02, except for the few species that may be synonymous with the same species mentioned above, other species of this genus have clustered into different separate clades (as shown in Figure 3).

#### 3.2.5 DNA barcode classification and identification of Tetraodontiformes

The classification and identification results based on DNA barcoding showed that 616 specimens were identified as 46 species, belonging to 23 genera, 6 families, and 4 Suborder (Table 1). Among them, although there are differences in morphological characteristics among the three groups of specimens of *T. rubripes*, *T. pseudommus*, and *T. chinensis*, they share the same haplotype, and their interspecific genetic distance ranges from 0.001 to 0.002 (average 0.002), far below the interspecific threshold of 0.02. In addition, in the NJ tree, they clustered into one clade. Therefore, *T. rubripes*, *T. pseudommus*, and *T. chinensis* should be synonymous with each other. According to the principle that the first published species name is the valid name, for the three species, *T. rubripes* should be their valid species name. Furthermore, this is also the case for *L. wheeleri* and *L. spadiceus* identified by morphological characteristics, which have the same haplotype and an interspecific genetic distance of 0.003, clustering into one clade in the NJ tree. Similarly, *L. wheeleri* and *L. spadiceus* should be synonymous, and *L. spadiceus* is the valid species name.

For *T. alboplumbeus* and *T. niphobles*, the DNA barcode identification results also showed that the two species may be synonymous. However, due to the fact that these 11 specimens identified as *T. alboplumbeus* do not completely have the morphological characteristics of *T. alboplumbeus*, it cannot be determined whether these specimens are hybrids of *T. alboplumbeus* and *T. niphobles*, or if *T. alboplumbeus* and *T. niphobles* are the same species, but they may undergo a morphological transformation process. More samples are needed to provide evidence for confirming the valid species names of these two species.

## 4 DISCUSSION

### 4.1 DNA barcoding threshold of Tetraodontiformes

DNA barcoding is used to identify species at molecular level, which is independent of time, region, and individual morphology (Hebert et al., 2003). This study shows that DNA barcoding can quickly and accurately identify the species of Tetraodontiformes, without being affected by key factors such as the integrity and development stage of the sample and the taxonomic expertise of the appraiser. For those closely related species, although the COI gene sequence may have high homology, they can still be distinguished by phylogenetic tree. For example, the sequence similarity between *T. flavidus* (Hap 100) and *T. bimaculatus* (Hap 91) has reached 98.7% (678 conserved sites in the 687 bp COI gene sequence), but the two species clustered into two independent clades in the NJ tree, thus enabling effective species identification.

This study indicates that species in *Takifugu* genus do have closer phylogenetic relationships than other reported species. The ratio of the average interspecific to intraspecific genetic distance is only 6.21 times. Among them, the largest interspecific genetic distance is between *T. oblongus* and *T. stictonotus*, with a value of 0.045; the smallest ones are *T. bimaculatus* and *T. flavidus*, with only 0.013; there are also multiple species with interspecific genetic distances <0.02 (as shown in Table 2). From this, it can be seen that the DNA barcode classification standard proposed by Hebert et al. (2004), with an interspecific genetic distance threshold of 0.02 and a ratio of interspecific to intra‐specific genetic distances greater than 10, is not entirely applicable to the species of *Takifugu* genus.

### 4.2 Quality control of sequences downloaded from databases

NCBI's GenBank and BOLD are the two most abundant databases of DNA barcode resources. In recent years, the number of DNA barcode sequences submitted to NCBI and BOLD has increased explosively. When we use these two databases for DNA barcode sequence alignment analysis, we sometimes encounter inaccurate matching results. It has been reported that there are many errors in the sequences of the NCBI database (Liu et al., 2020; Shen et al., 2013). Some studies believe that the BOLD database has conducted a more rigorous review and screening on the submitted sequences, so the data in this database is more reliable (Macher et al., 2017; Wang et al., 2009). In fact, there are also wrong sequences in the BOLD database (Lis et al., 2016).

In this study, all sequences were run BLAST in NCBI and BOLD databases. The Blast results showed that most of the query sequences were able to match the target species with the highest identities in the database, which was consistent with the morphological identification results. But there were also some unexpected matching results of the query sequences, with multiple highly similar target species, some of which were consistent with morphological identification, while others were inconsistent. For instance, the query sequence of the *T. porphyreus* specimen was matched to the *T. porphyreus*, and in addition, two COI gene sequences of *T. porphyreus* (KT951818, KT951819) in the database were also matched, with identities of 100%. But apart from these two sequences, it could not match with other sequences of *T. porphyreus* in the database. Therefore, the information on those two sequences in the database is inaccurate. In addition, the query sequences of *T. chinensis*, *T. pseudommus*, and *T. rubripes* showed 99.56% identities to the two mitochondrial genome sequences (NC_024199, KJ562276) of *T. flavidus* in BOLD and NCBI. After checking, it was found that the information of these two sequences were also incorrect. Therefore, there are some incorrect sequences of Tetraodontiformes in the NCBI and BOLD databases.

The unreliable data in the databases will directly lead to the misidentification of species. To reduce the interference of the wrong sequence in the database on the analysis results, it is suggested that the relevant sequences in the database should be strictly screened when using DNA barcoding technology to identify species. For instance, when screening DNA barcode sequences, we can select sequences uploaded by at least three different authors, or select the sequences that have already been published and provide morphological characteristics of the species. If the information in NCBI and BOLD databases is found to be inaccurate when the voucher specimens with complete morphological characteristics are available, the database or data submitter shall be contacted in time to correct the sequence information. In this study, DNA barcoding technology and morphological methods were used synchronously to identify species of Tetraodontiformes, which could ensure the accuracy of species identification. Therefore, the DNA barcode sequences of Tetraodontiformes provided in this study can be used as reference sequences for the identification of the species in this order.

### 4.3 Synonym phenomenon in the order of Tetraodontiformes

Early taxonomic research was mainly based on morphological characteristics. Due to the fact that the morphological characteristics of fish are not only susceptible to subjective factors but also undergo significant change at different developmental stages and environmental conditions, coupled with delayed information exchange among taxonomists in different regions, the same species may have two or more different Latin names, which is also called synonym.

This study shows that there are a lot of synonyms in Tetraodontiformes. For example, *M. leiurus* is the synonym of *P. leiurus*, but the samples morphologically identified as *P. leiurus* were identified as *M. leiurus* in NCBI, and there is no information about *P. leiurus* can be found in NCBI. What's more, the samples which morphologically identified as *D. nigroviridis*, in NCBI and BOLD databases, were identified as *T. nigroviridis*. In fact, in the FishBase *T. nigroviridis* is the synonym of *D. nigroviridis*. The variety of species names in the database will affect the molecular identification results of species. We consider that the unsynchronized information in the database is also one of the reasons for the confusion of species identification of Tetraodontiformes.

In addition, in the FishBase, both *Lagocephalus wheeleri* (Abe, Tabeta & Kitahama, 1984) and *Lagocephalus spadiceus* (Richardson, 1845) are effective species, and they can be distinguished from each other in terms of morphological characteristics. For example, the former has elliptical dorsal spinule patch, while the latter has a rhomboidal patch with a posterior extension. However, some studies believe that these morphological features cannot be used to distinguish the two, and *L. wheeleri* may be a synonym for *L. spadiceus* (Matsuura, 2010; Sakai et al., 2021). In this study, the results of haplotype analysis showed that they had the same haplotype and clustered into the same clade in neighbor‐joining tree. Additionally, their interspecific genetic distance was only 0.003. This shows, the molecular classification results support that *L. wheeleri* is synonymous with *L. spadiceus*.

Moreover, there are many reports believe that *T. rubripes*, *T. chinensis* and *T. pseudommus* may be different phenotypes of the same species, that is, *T. chinensis* and *T. pseudommus* are the synonyms of *T. rubripes* (Cui et al., 2005; Liu et al., 1999; Park et al., 2020; Reza et al., 2008; Song et al., 2001). However, all three species are valid in Fishbase and can be distinguished by morphological characteristics. *T. rubripes* has irregular round black spots and white patterns in front of the caudal fin on the sides of the body, *T. chinensis* has no such black marks, while *T. pseudommus* has white spots scattered on a black background on the dorsal and lateral sides of the body (Baek et al., 2018; Reza et al., 2008). In this study, the initial morphological identification results showed that eight samples were identified as T. chinensis, 4 as *T. pseudommus*, and 27 as *T. rubripes*. However, further research has found that samples identified by morphology as the three species had the same haplotype, and the interspecific genetic distance among them ranged from 0.001 to 0.002. In additional, in the NJ tree, they clustered into one clade. To sum up, the *T. chinensis* and *T. pseudommus* are the synonyms of *T. rubripes*, and *T. rubripes* is the valid name.

And the morphologic features of *T. alboplumbeus* and *T. niphobles* are so similar that they are difficult to distinguish. It is generally believed that *T. niphobles* have obvious chest spots, while the chest spots of *T. alboplumbeus* are not obvious, and there are several dark bands on the back of *T. alboplumbeus*. In this study, the DNA barcode identification results also showed that the two species may be synonyms. However, since the 11 specimens identified as *T. alboplumbeus* do not completely have the morphological characteristics of *T. alboplumbeus*, it is not certain that these specimens are hybrids of *T. alboplumbeus* and *T. niphobles*. Or that *T. alboplumbeus* and *T. niphobles* are the same species, but they will undergo a morphological transformation process. Matsuura (2017) regarded *T. niphobles* and *T. alboplumbeus* as synonyms based on the same color patterns and no differences in morphological characteristics between the type specimens. Some researchers support this view as well (Okabe et al., 2019), and these two species are also considered to be synonyms in NCBI. However, in the FishBase, it is considered that both *T. alboplumbeus* and *T. niphobles* are effective species. Zhang and He (2008) showed that 12 s and cytb genes could separate *T. alboplumbeus* and *T. niphobles*, but the study did not describe the morphological characteristics of the two species. Zhou et al. (2020) showed that the mitochondrial genome can separate *T. alboplumbeus* and *T. niphobles* effectively. However, the study did not describe the morphological characteristics of the two species as well. Therefore, whether *T. alboplumbeus* and *T. niphobles* are synonyms has not been determined.

### 4.4 Reconstruction of Tetraodontiformes phylogenetic relationships

Many researchers have studied the relationship between families of Tetraodontiformes based on different methods (Alfaro & Brock, 2007; Breder Jr & Clark, 1947; Holcroft, 2005; Leis, 1984; Rosen, 1984; Santini & Tyler, 2010; Tyler & Sorbini, 1996; Winterbottom, 1974; Yamanoue et al., 2007, 2008). In their study, there were more or less differences in the phylogenetic tree of Tetraodontiformes. The commonality is that they unanimously agreed that Balistidae and Monacanthidae were closely related, and Diodontidae and Tetraodontidae were closely related as well.

In this study, the Neighbor‐joining tree based on the haplotype of COI gene of Tetraodontiformes shows that the species of six families are split into two branches. And Diodontidae and Tetraodontidae clustered into a branch, Monacanthidae, Balistidae, Ostraciidae, and Triacanthidae clustered into another branch, as shown in Figure 3. The result that Diodontidae and Tetraodontidae are clustered in a separate branch is consistent with the research results of others. However, unlike other studies, Balistidae and Monacanthidae did not cluster into a branch in this study. The reason for this result may be that there are not enough samples collected in this study. For example, only one sample of Triacanthidae was collected. It is also possible that the COI gene sequence is not suitable for the systematic relationship study of families and higher taxonomic levels.

In the Neighbor‐joining tree based on the haplotype of COI gene of Tetraodontiformes, the species of the same genus clustered together, and the same species are clustered into a clade, which is basically consistent with the results of morphological identification. Although the interspecific genetic distance of some species of Takifugu is <0.02 (as shown in Table 2), in the Neighbor‐joining tree, except for *T. pseudommus*, *T. rubripes*, and *T. chinensis*, each species can gather into a separate clade. It indicates that the COI gene sequence is suitable for the classification and identification of genera and lower taxonomic levels.

## 5 CONCLUSION

In this study, 616 samples of Tetraodontiformes were identified by using morphological characteristics and DNA barcoding technology. It revealed that DNA barcoding can be effectively used in the identification of Tetraodontiformes.

Moreover, it is suggested that DNA barcoding can provide molecular evidence for clarifying the problem of species synonyms and confirming valid species names. It provided molecular evidence for clarifying the valid species names of *L. spadiceus* and *T. rubripes*.

In addition, this study reconfirms that there are some incorrect sequences in both NCBI and BOLD databases. It is suggested that synonyms and unreliable data are also the reasons for the confusion of taxonomic identification of Tetraodontiformes. We suggest that when using DNA barcode technology to identify species, the sequence in the database needs to be strictly screened. When the sample has complete morphological characteristics, the final identification results of species should be determined by combining the morphological characteristics.

## AUTHOR CONTRIBUTIONS

**Kaiying Liu:** Data curation (lead); formal analysis (lead); investigation (lead); methodology (lead); resources (equal); validation (lead); visualization (lead); writing – original draft (lead); writing – review and editing (equal). **Hongyue Sun:** Data curation (equal); investigation (equal); resources (equal); writing – original draft (equal). **Xin Zhao:** Investigation (equal). **Chaoqun Wang:** Investigation (equal); resources (equal). **Changting An:** Writing – review and editing (equal). **Ang Li:** Writing – review and editing (equal). **Shufang Liu:** Conceptualization (lead); data curation (equal); formal analysis (equal); funding acquisition (supporting); investigation (equal); methodology (equal); project administration (equal); resources (lead); supervision (lead); validation (equal); visualization (equal); writing – original draft (equal); writing – review and editing (equal). **Zhimeng Zhuang:** Conceptualization (equal); supervision (equal).

## CONFLICT OF INTEREST STATEMENT

We wish to draw the attention of the Editor to the following facts may be considered as potential conflicts of interest and significant financial contributions to this work. All authors agreed to this submission and the corresponding author has been authorized by co‐authors. This manuscript has not been published before and is not concurrently being considered for publication elsewhere. This manuscript does not violate any copyright or other personal proprietary right of any person or entity and it contains no abusive, defamatory, obscene, or fraudulent statements, nor any other statements that are unlawful in any way.

## Data Availability

DNA sequences have been deposited in GenBank under Accession numbers OQ700230–OQ700845. Details regarding individual samples are available in Table 1.
